# Factors associated with prolonged postoperative hospital stay after cholecystectomy for acute cholecystitis in a Peruvian emergency hospital

**DOI:** 10.3389/fsurg.2026.1921693

**Published:** 2026-10-14

**Authors:** Yudi N. Tolentino-Alania, Fiorella A. Sanchez-Ubaldo, Miguel A. Arce-Huamani

**Affiliations:** 1 Programa Académico de Medicina Humana, Facultad de Ciencias de la Salud, Universidad Privada San Juan Bautista, Lima, Peru; 2 Programa Académico de Medicina Humana, Facultad de Ciencias de la Salud, Universidad Privada Norbert Wiener, Lima, Peru

**Keywords:** acute cholecystitis, cholecystectomy, emergency surgery, length of stay, postoperative complications, SDG 3: Good Health and Well-Being

## Abstract

**Background:**

Prolonged postoperative hospital stay after cholecystectomy for acute cholecystitis may reflect baseline vulnerability and disease severity. We assessed preoperative factors associated with prolonged postoperative stay in a Peruvian emergency hospital.

**Methods:**

We conducted a retrospective cohort study of patients undergoing cholecystectomy for acute cholecystitis in 2024. All potentially eligible source records were screened. Prolonged postoperative stay was defined as >3 days from surgery to discharge. Adjusted risk ratios (aRRs) and 95% confidence intervals (CIs) were estimated using modified Poisson regression with robust variance in a prespecified model restricted to preoperative variables.

**Results:**

Of 540 records screened, 422 patients were eligible. Median postoperative stay was 2.4 days (IQR, 1.0–3.8), and 158 patients (37.4%) stayed >3 days. Higher risk was observed for age 45–64 years (aRR, 1.51; 95% CI, 1.09–2.10), age ≥65 years (aRR, 1.95; 95% CI, 1.37–2.76), diabetes mellitus (aRR, 1.31; 95% CI, 1.02–1.67), and ASA IV–V status (aRR, 1.50; 95% CI, 1.04–2.16). Hypertension and active smoking showed inverse associations and require cautious interpretation. Postoperative complications occurred in 111 patients (26.3%) and were analyzed descriptively.

**Conclusion:**

Older age, diabetes mellitus, and higher preoperative ASA status were associated with prolonged postoperative stay. These findings warrant prospective validation and should not be interpreted as causal.

## 1 Introduction

Acute cholecystitis remains one of the most frequent biliary emergencies requiring surgical care, and its clinical relevance is increasing because gallbladder and biliary tract diseases continue to generate a substantial global burden (1). A recent systematic review estimated that gallbladder stones affect 5.86% of the general population worldwide, with a higher pooled prevalence in Latin America and the Caribbean of 15.43% (2). In Colombia, an analysis of national health records identified 343,254 cases of acute cholecystitis between 2018 and 2022, corresponding to a prevalence of 681 cases per 100,000 inhabitants and a predominance among women (3). Acute calculous cholecystitis is therefore not only a common complication of gallstone disease but also a condition that places sustained pressure on emergency surgical services (4).

Early laparoscopic cholecystectomy is currently recommended as the preferred treatment for acute calculous cholecystitis when patients are suitable surgical candidates, because it provides definitive source control during the index admission (5). The Tokyo Guidelines 2018 standardized diagnostic criteria and severity grading, allowing clinicians to classify patients according to systemic inflammation, local findings, imaging results, organ dysfunction, and operative risk (6). These guidelines also proposed management flowcharts that integrate severity grade, comorbidity burden, surgical risk, and institutional expertise when deciding between early surgery, gallbladder drainage, or alternative strategies (7). In difficult laparoscopic cholecystectomy, the same guideline framework emphasizes safe operative steps, including bailout procedures when the critical view of safety cannot be achieved (8). Appropriate antimicrobial therapy remains essential in the initial management of biliary infection, although antibiotics alone do not replace timely surgical or drainage-based source control in selected cases (9).

Despite these recommendations, prolonged hospital stay after surgery remains clinically important because it reflects not only disease severity but also operative complexity, postoperative recovery, and hospital resource use. In an acute-care surgical setting, longer preoperative waiting time was associated with longer length of stay among patients treated with laparoscopic cholecystectomy (10). A retrospective cohort applying TG18 found that patients operated beyond seven days from symptom onset had longer operative time than those operated earlier, suggesting greater technical difficulty with delayed surgery (11). Emergency cholecystectomy may shorten total hospitalization, although some studies have reported trade-offs in perioperative morbidity when compared with delayed surgery (12). Other evidence suggests that laparoscopic cholecystectomy can remain feasible even when symptoms exceed the traditional 72-hour window, provided that patient selection and surgical expertise are adequate (13).

Patient-related and intraoperative factors may further explain why some patients require longer postoperative hospitalization. A systematic review found that increasing age was associated with longer length of stay after laparoscopic cholecystectomy (14), and a recent cohort confirmed that older adults undergoing urgent laparoscopic cholecystectomy for acute cholecystitis had longer hospitalization and higher costs (15). Conversion from laparoscopic to open surgery, reported in 2%–15% of laparoscopic cholecystectomies, has been associated with emergency treatment, acute inflammation, adhesions, older age, and longer postoperative recovery (16). Drain placement and converted procedures have also been linked to prolonged hospitalization, even when postoperative complications are not clearly reduced (17). Because emergency laparoscopic cholecystectomy pathways may reduce waiting times and improve service delivery, identifying factors associated with prolonged postoperative stay is particularly relevant in high-demand emergency hospitals (18).

Therefore, this study aimed to determine the preoperative factors associated with prolonged postoperative hospital stay among patients undergoing cholecystectomy for acute cholecystitis in a Peruvian emergency hospital.

## 2 Materials and methods

### 2.1 Study design and setting

We conducted a retrospective cohort study of patients who underwent cholecystectomy for acute cholecystitis at Hospital de Emergencias José Casimiro Ulloa, a public emergency hospital in Lima, Peru. Patients were identified from institutional clinical records between January and December 2024, and their clinical course was retrospectively reconstructed from hospital admission through surgery and discharge during the index hospitalization. For the primary analysis, time zero was defined as the recorded date and time of surgery, and the outcome period extended from surgery to hospital discharge. No prospective intervention or patient contact was performed.

The study was designed and reported in accordance with the Strengthening the Reporting of Observational Studies in Epidemiology (STROBE) Statement. The completed STROBE checklist is provided as Supplementary File S1.

### 2.2 Study population and participant selection

The source dataset comprised 540 hospital records identified from the institutional registry as potentially eligible for the study during the January–December 2024 study period. For the revised analysis, all source records were screened and no sampling procedure was applied.

After application of the predefined eligibility criteria, 118 records were excluded: 28 because no cholecystectomy was performed during the index admission, 26 because key timestamps or the length-of-stay outcome were incomplete, 20 because acute cholecystitis was not confirmed after chart review, 18 because the clinical record was incomplete for the main covariates, 12 because the procedure was an elective or interval cholecystectomy, eight because the patient was younger than 18 years, and six because they represented duplicate index episodes. The final retrospective cohort therefore included all 422 eligible patients. Participant selection is shown in Figure 1.

**Figure 1:**
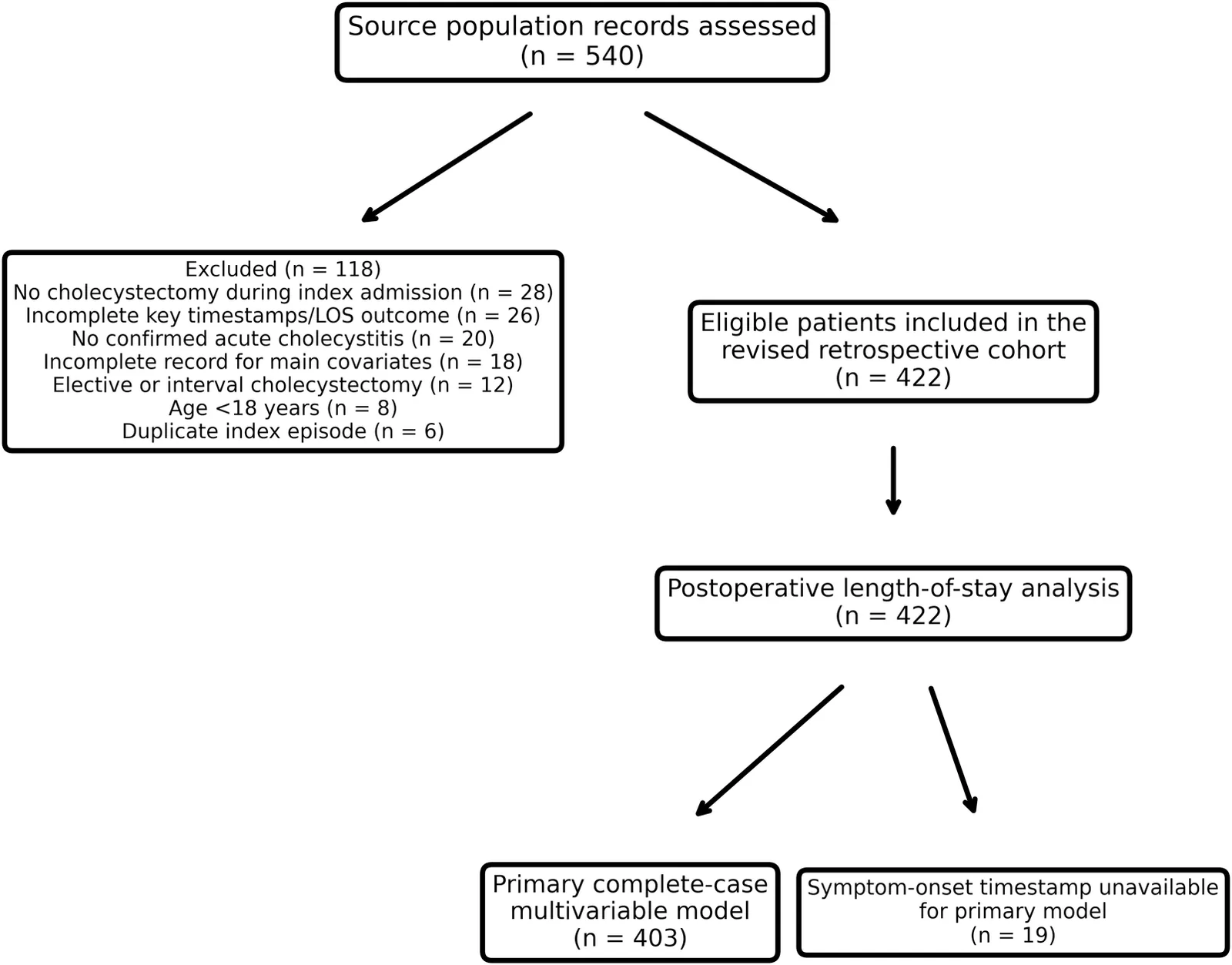
Participant flow through the study. A total of 540 source-population records were assessed for eligibility. After exclusion of 118 records, 422 eligible patients were included in the revised retrospective cohort and postoperative length-of-stay analysis. The primary complete-case multivariable analysis included 403 patients because the symptom-onset timestamp was unavailable for 19 patients.

### 2.3 Eligibility criteria

Patients were eligible if they were aged ≥18 years, had acute cholecystitis confirmed after review of the clinical record, underwent cholecystectomy during the index hospitalization, and had sufficient information to determine postoperative length of stay and the main study covariates. Records were excluded when acute cholecystitis was not confirmed, cholecystectomy was not performed during the index admission, the procedure corresponded to an elective or interval cholecystectomy, essential timestamps or covariate data were unavailable, the patient was younger than 18 years, or the record represented a duplicate index episode.

### 2.4 Variables and definitions

The primary outcome was prolonged postoperative hospital stay. Postoperative length of stay was calculated from the recorded date and time of surgery to the date and time of hospital discharge and was expressed in days. A prolonged postoperative hospital stay was defined as >3 days, whereas a postoperative stay of ≤3 days was considered non-prolonged. The >3-day threshold was selected *a priori* based on previous literature using postoperative stay beyond 3 days as a marker of a complicated postoperative course following laparoscopic cholecystectomy (19). The admission-to-surgery interval was calculated separately from the recorded admission and surgery timestamps and expressed in hours. Total hospital stay, calculated from admission to discharge, was retained as a descriptive measure but was not used as the primary outcome.

The prespecified preoperative explanatory variables included age group, sex, hypertension, diabetes mellitus, obesity, active smoking, American Society of Anesthesiologists (ASA) physical status, Tokyo Guidelines 2018 severity grade, and time from symptom onset to surgery. Age was categorized as 18–44, 45–64, and ≥65 years. Hypertension and diabetes mellitus were recorded as separate binary variables. Obesity was defined as a body mass index ≥30 kg/m^2^. Active smoking was categorized as yes or no, and ASA physical status was grouped as ASA I–II, ASA III, and ASA IV–V. Acute cholecystitis severity was classified according to the Tokyo Guidelines 2018 as Grade I, Grade II, or Grade III.

Time from symptom onset to surgery was calculated from the documented symptom-onset and surgery timestamps and expressed in hours; for regression analysis, it was modeled continuously per 24-h increase.

Intraoperative characteristics, including initial surgical approach, operative time, and conversion from laparoscopic to open surgery, were described separately and were not included in the primary explanatory model. Postoperative complications were considered postoperative outcomes rather than predictors because they occurred after surgery during the same admission in which postoperative length of stay was measured. Surgical site infection, pneumonia, sepsis, mechanical ventilation, and deep vein thrombosis were recorded as separate binary variables (yes/no) based on diagnoses documented by the treating clinical team during the index hospitalization. Because each complication was coded independently, co-occurring postoperative complications could be recorded simultaneously when documented in the same patient.

Surgical site infection was defined as a documented postoperative infection involving the surgical site, pneumonia as a documented postoperative diagnosis of pneumonia, and sepsis as a documented postoperative diagnosis of sepsis. Because the retrospective records did not consistently contain sufficient clinical detail for independent adjudication using standardized surveillance criteria, these diagnoses were accepted as recorded in the medical record. Secondary postoperative outcomes included in-hospital mortality and 30-day readmission. Thirty-day readmission was evaluated among patients discharged alive from the index hospitalization.

### 2.5 Data collection procedures

After institutional and ethics approvals were obtained, data were retrospectively extracted from clinical records using a structured data collection form. Recorded dates and times of hospital admission, symptom onset when documented, surgery, and discharge were extracted and used to calculate the symptom-onset-to-surgery interval, admission-to-surgery interval, postoperative length of stay, and total hospital stay.

Preoperative data included age, sex, hypertension, diabetes mellitus, body mass index, active smoking, ASA physical status, and Tokyo Guidelines 2018 severity grade. Intraoperative information included the initial surgical approach, operative time, and conversion from laparoscopic to open surgery. Postoperative information included surgical site infection, pneumonia, sepsis, mechanical ventilation, deep vein thrombosis, in-hospital mortality, and 30-day readmission. The exact date and time of onset or diagnosis of postoperative complications relative to surgery were not consistently available in the source records and therefore could not be reliably classified according to whether they occurred before or after postoperative day 3. For this reason, postoperative complications were not included as predictors in the primary regression model.

The database underwent coding and consistency checks before analysis to identify missing, duplicated, or implausible values. No statistical imputation was performed.

### 2.6 Statistical analysis

Categorical variables were summarized using absolute frequencies and percentages. Continuous variables with asymmetric distributions were summarized using medians and interquartile ranges (IQRs). These included the admission-to-surgery interval, symptom-onset-to-surgery interval, postoperative length of stay, and total hospital stay.

Crude and adjusted risk ratios (RRs) with 95% confidence intervals (CIs) for prolonged postoperative hospital stay were estimated using modified Poisson regression with robust variance. The multivariable adjustment set was prespecified on clinical and temporal grounds rather than selected according to univariable statistical significance. Covariates were chosen because they represented baseline demographic characteristics, comorbidity burden, preoperative physiological status, acute cholecystitis severity, or timing of presentation that could plausibly influence both perioperative clinical course and postoperative recovery. The prespecified model therefore included age group, sex, hypertension, diabetes mellitus, obesity, active smoking, ASA physical status, Tokyo Guidelines 2018 severity grade, and time from symptom onset to surgery.

Postoperative complications and intraoperative characteristics were not included as explanatory variables in the primary model because they occurred after the preoperative assessment and could introduce temporal ambiguity or represent intermediate events. Postoperative complications, in-hospital mortality, and 30-day readmission were therefore summarized descriptively.

Time from symptom onset to surgery was analyzed continuously per 24-h increase. Because symptom-onset information was unavailable for 19 patients, the primary multivariable analysis was restricted to complete cases (*n* = 403), and no imputation was performed. As a sensitivity analysis, the primary multivariable model was repeated using a more stringent definition of prolonged postoperative hospital stay (>4 days) to assess the robustness of the findings to the selected >3-day threshold. The same prespecified covariate set and complete-case approach were applied. Consistency in the direction and magnitude of the principal associations across the primary and sensitivity models was used to assess the robustness of the main findings to the outcome definition. Multicollinearity among model covariates was assessed using variance inflation factors (VIFs), which ranged from 1.02 to 1.88, indicating no relevant multicollinearity.

All analyses were performed using Stata version 19. Statistical significance was evaluated using two-sided *p*-values <0.05.

## 3 Results

A total of 540 source-population records were screened, of which 422 patients met the eligibility criteria and were included in the retrospective cohort (Figure 1). Median postoperative hospital stay was 2.4 days (IQR, 1.0–3.8 days), while median total hospital stay was 3.5 days (IQR, 2.4–5.0 days). The median admission-to-surgery interval was 27.4 h (IQR, 18.0–38.6 h). Prolonged postoperative hospital stay, defined as >3 days after surgery, occurred in 158 patients (37.4%).

The median age was 53 years (IQR, 37–65 years), and 247 patients (58.5%) were male. Hypertension was present in 165 patients (39.1%), diabetes mellitus in 124 (29.4%), and obesity, defined as BMI ≥30 kg/m^2^, in 127 (30.1%). Regarding preoperative clinical status, 267 patients (63.3%) were classified as ASA I–II, 117 (27.7%) as ASA III, and 38 (9.0%) as ASA IV–V. According to the Tokyo Guidelines 2018, 173 patients (41.0%) had Grade I, 177 (41.9%) Grade II, and 72 (17.1%) Grade III acute cholecystitis. Symptom onset was documented in 403 patients (95.5%), with a median symptom-onset-to-surgery interval of 72.2 h (IQR, 55.2–96.1 h) (Table 1).

**Table 1:** Patient and perioperative characteristics of patients undergoing cholecystectomy for acute cholecystitis.

Variable	Overall (*N* = 422)
Postoperative hospital stay, days, median (IQR)	2.4 (1.0–3.8)
Total hospital stay, days, median (IQR)	3.5 (2.4–5.0)
Admission-to-surgery interval, h, median (IQR)	27.4 (18.0–38.6)
Prolonged postoperative hospital stay >3 days	158 (37.4)
Age, years, median (IQR)	53 (37–65)
Age group	
18–44 years	150 (35.5)
45–64 years	166 (39.3)
≥65 years	106 (25.1)
Sex	
Male	247 (58.5)
Female	175 (41.5)
Hypertension	165 (39.1)
Diabetes mellitus	124 (29.4)
BMI, kg/m^2^, median (IQR)	27.6 (25.4–30.6)
Obesity, BMI ≥30 kg/m^2^	127 (30.1)
Active smoking	104 (24.6)
ASA classification	
ASA I–II	267 (63.3)
ASA III	117 (27.7)
ASA IV–V	38 (9.0)
Tokyo Guidelines 2018 severity grade	
Grade I	173 (41.0)
Grade II	177 (41.9)
Grade III	72 (17.1)
Symptom onset documented	403 (95.5)
Symptom onset-to-surgery interval, h, median (IQR)^a^	72.2 (55.2–96.1)
Initial surgical approach	
Laparoscopic	389 (92.2)
Open	33 (7.8)
Conversion to open surgery among laparoscopic starts^b^	54/389 (13.9)
Operative time, min, median (IQR)	111 (85–146)
Operative time ≥2 h	179 (42.4)

Values are presented as *n* (%) unless otherwise indicated. ASA, American Society of Anesthesiologists; BMI, body mass index; IQR, interquartile range.

a Symptom onset-to-surgery interval was available for 403 patients.

b The conversion rate was calculated among the 389 procedures that began laparoscopically.

Overall, 389 procedures (92.2%) began laparoscopically and 33 (7.8%) began as open procedures. Among procedures initiated laparoscopically, 54 of 389 (13.9%) were converted to open surgery. Median operative time was 111 min (IQR, 85–146 min), and 179 procedures (42.4%) lasted ≥2 h (Table 1).

Postoperative complications were analyzed as descriptive outcomes rather than explanatory variables. Overall, 111 patients (26.3%) experienced at least one postoperative complication. Surgical site infection occurred in 47 patients (11.1%), pneumonia in 26 (6.2%), and sepsis in 20 (4.7%). Five patients (1.2%) died during the index admission. Among the 417 patients discharged alive, 33 (7.9%) were readmitted within 30 days. These outcomes are summarized according to postoperative length of stay in Table 2.

**Table 2:** Postoperative complications and short-term outcomes according to postoperative length of stay.

Postoperative outcome	Overall	Postoperative stay ≤3 days	Postoperative stay >3 days
Any postoperative complication	111 (26.3)	53 (20.1)	58 (36.7)
Surgical site infection	47 (11.1)	23 (8.7)	24 (15.2)
Pneumonia	26 (6.2)	11 (4.2)	15 (9.5)
Sepsis	20 (4.7)	10 (3.8)	10 (6.3)
Mechanical ventilation	9 (2.1)	7 (2.7)	2 (1.3)
Deep vein thrombosis	9 (2.1)	2 (0.8)	7 (4.4)
In-hospital mortality	5 (1.2)	3 (1.1)	2 (1.3)
30-day readmission^a^	33/417 (7.9)	19/261 (7.3)	14/156 (9.0)

Values are presented as *n* (%) and are descriptive only. No inferential comparisons were performed because postoperative complications occurred after surgery during the same admission in which postoperative length of stay was measured.

a Thirty-day readmission was assessed among the 417 patients discharged alive; the five patients who died during the index admission were not at risk for readmission.

The primary complete-case multivariable analysis included 403 patients. Compared with patients aged 18–44 years, those aged 45–64 years had a higher risk of prolonged postoperative hospital stay [adjusted RR (aRR), 1.51; 95% CI, 1.09–2.10; *p* = 0.014], as did those aged ≥65 years (aRR, 1.95; 95% CI, 1.37–2.76; *p* < 0.001). Diabetes mellitus was also associated with a higher risk (aRR, 1.31; 95% CI, 1.02–1.67; *p* = 0.031), as was ASA IV–V status compared with ASA I–II (aRR, 1.50; 95% CI, 1.04–2.16; *p* = 0.031). Hypertension (aRR, 0.72; 95% CI, 0.55–0.95; *p* = 0.019) and active smoking (aRR, 0.68; 95% CI, 0.48–0.95; *p* = 0.025) showed inverse associations with prolonged postoperative stay. Sex, obesity, ASA III status, Tokyo Guidelines 2018 Grade II or III severity, and time from symptom onset to surgery were not independently associated with the outcome after adjustment (Table 3). VIFs ranged from 1.02 to 1.88, indicating no relevant multicollinearity. Adjusted estimates are displayed in Figure 2.

**Table 3:** Crude and adjusted risk ratios for prolonged postoperative hospital stay.

Variable	Category	Crude RR (95% CI)	*p*-value	Adjusted RR (95% CI)	*p*-value
Age group	18–44 years	Reference	–	Reference	–
	45–64 years	1.57 (1.12–2.20)	0.009	**1.51** **(1.09–2.10)**	**0** **.** **014**
	≥65 years	1.98 (1.41–2.78)	<0.001	**1.95** **(1.37–2.76)**	**<0** **.** **001**
Sex	Female	Reference	–	Reference	–
	Male	1.06 (0.82–1.37)	0.638	1.11 (0.87–1.43)	0.404
Hypertension	No	Reference	–	Reference	–
	Yes	0.79 (0.60–1.03)	0.085	**0.72** **(0.55–0.95)**	**0** **.** **019**
Diabetes mellitus	No	Reference	–	Reference	–
	Yes	1.30 (1.01–1.68)	0.041	**1.31** **(1.02–1.67)**	**0** **.** **031**
Obesity (BMI ≥30 kg/m^2^)	No	Reference	–	Reference	–
	Yes	1.07 (0.82–1.39)	0.642	1.04 (0.80–1.34)	0.794
Active smoking	No	Reference	–	Reference	–
	Yes	0.67 (0.47–0.94)	0.022	**0.68** **(0.48–0.95)**	**0** **.** **025**
ASA classification	ASA I–II	Reference	–	Reference	–
	ASA III	1.31 (1.00–1.72)	0.048	1.24 (0.95–1.62)	0.110
	ASA IV–V	1.53 (1.06–2.23)	0.025	**1.50** **(1.04–2.16)**	**0** **.** **031**
Tokyo Guidelines 2018 severity	Grade I	Reference	–	Reference	–
	Grade II	1.34 (1.02–1.78)	0.038	1.06 (0.78–1.43)	0.709
	Grade III	1.18 (0.81–1.72)	0.379	0.75 (0.48–1.17)	0.204
Symptom onset to surgery	Per 24-h increase	1.10 (1.02–1.20)	0.013	1.09 (0.99–1.20)	0.071

RR, risk ratio; CI, confidence interval; ASA, American Society of Anesthesiologists; BMI, body mass index. Risk ratios were estimated using modified Poisson regression with robust variance. The primary multivariable model was prespecified on clinical and temporal grounds, independent of univariable *p*-values, and included all variables shown in the table. The adjusted analysis included 403 patients with complete symptom-onset data. Postoperative complications and intraoperative characteristics were not included in the primary explanatory model. Variance inflation factors ranged from 1.02 to 1.88, indicating no relevant multicollinearity.

Bold values indicate statistically significant associations in the adjusted model (*p* < 0.05).

**Figure 2:**
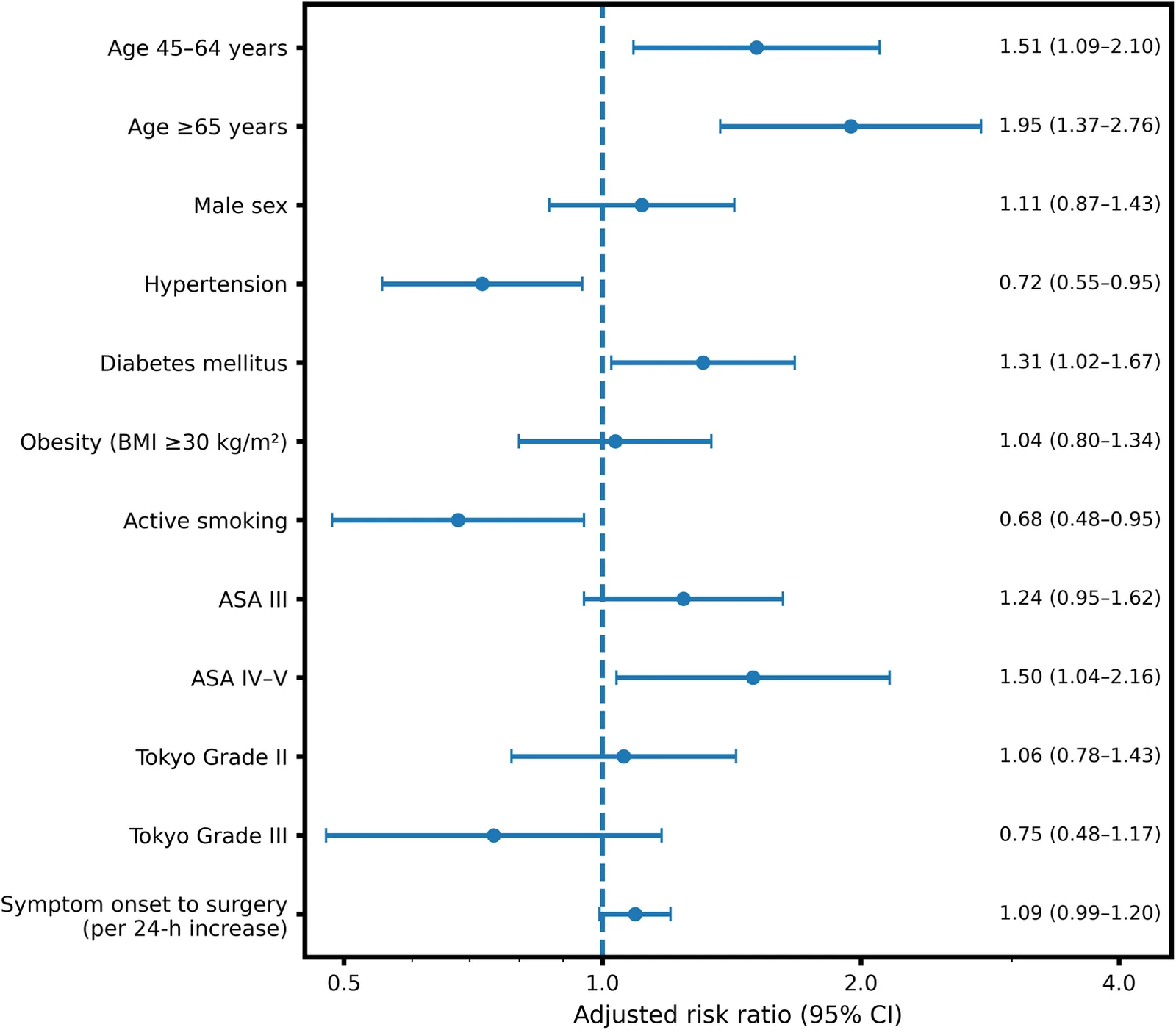
Adjusted risk ratios for prolonged postoperative hospital stay among patients undergoing cholecystectomy for acute cholecystitis. Points represent adjusted risk ratios and horizontal lines represent 95% confidence intervals. Estimates were obtained from a prespecified modified Poisson regression model with robust variance restricted to preoperative variables. The model included age group, sex, hypertension, diabetes mellitus, obesity, active smoking, ASA physical status, Tokyo Guidelines 2018 severity grade, and time from symptom onset to surgery. The analysis included 403 patients with complete data. The vertical dashed line indicates RR = 1. ASA, American Society of Anesthesiologists; BMI, body mass index; CI, confidence interval; RR, risk ratio.

In the sensitivity analysis using a more stringent threshold of >4 postoperative days, 95 of 422 patients (22.5%) met the alternative definition of prolonged stay. The main pattern of associations was broadly preserved: age ≥65 years (aRR, 2.44; 95% CI, 1.49–4.01), diabetes mellitus (aRR, 1.50; 95% CI, 1.05–2.15), and ASA IV–V status (aRR, 2.15; 95% CI, 1.37–3.39) remained associated with prolonged postoperative stay. Full estimates are provided in Supplementary Table S1.

## 4 Discussion

In this retrospective cohort of patients undergoing cholecystectomy for acute cholecystitis, prolonged postoperative hospital stay occurred in 37.4% of patients. After adjustment for prespecified preoperative factors, older age, diabetes mellitus, and ASA IV–V physical status were associated with a higher risk of postoperative stay exceeding three days. Hypertension and active smoking showed inverse associations, although these findings require cautious interpretation. Tokyo Guidelines 2018 severity grade and time from symptom onset to surgery did not remain independently associated with prolonged postoperative stay. Importantly, intraoperative characteristics and postoperative complications were not treated as predictors in the primary model because of their temporal position relative to the preoperative assessment and postoperative outcome. The associations of age ≥65 years, diabetes mellitus, and ASA IV–V status were also preserved when prolonged postoperative stay was redefined using a more stringent >4-day threshold, supporting the robustness of the principal findings to the outcome cutoff.

Median postoperative hospital stay was 2.4 days, whereas the median admission-to-surgery interval was 27.4 h. Separating these periods was particularly important in an emergency-care setting because preoperative waiting may substantially influence total hospital length of stay. Bressan et al. (10) similarly reported that longer preoperative waiting was associated with longer total hospitalization among patients undergoing laparoscopic cholecystectomy in an acute-care surgical setting. Zeineddin et al. (19) used a comparable postoperative threshold, defining a complicated postoperative course partly on the basis of postoperative stay exceeding three days. These findings support the use of postoperative rather than total hospital stay when the objective is to evaluate recovery after surgery.

Age showed one of the clearest associations in our cohort. Patients aged 45–64 years and those aged ≥65 years had higher adjusted risks of prolonged postoperative stay than patients aged 18–44 years, with the strongest association observed in the oldest group. Lee et al. (20) reported progressively worse surgical outcomes with advancing age in patients with acute cholecystitis, while Kao et al. (15) found that older age was associated with longer hospitalization and higher costs following urgent laparoscopic cholecystectomy. Older patients may have lower physiological reserve and a greater burden of comorbidity, frailty, and systemic inflammation, factors that may contribute to slower postoperative recovery. However, age should be interpreted as a marker of vulnerability rather than a causal determinant in isolation.

Diabetes mellitus remained associated with prolonged postoperative stay after multivariable adjustment, whereas obesity was not associated with the outcome after adjustment. Previous studies have linked diabetes to increased perioperative morbidity in acute cholecystitis (21), and diabetes and obesity have also been associated with surgical site infection following cholecystectomy (22). High BMI has additionally been reported among factors related to technically difficult laparoscopic cholecystectomy (23). The absence of an independent association for obesity in our cohort suggests that BMI alone may not adequately capture metabolic or operative risk once age, diabetes, ASA status, disease severity, and symptom duration are considered.

ASA IV–V status remained associated with a higher risk of prolonged postoperative stay, whereas ASA III did not retain an independent association after adjustment. Osterman et al. (24) reported increasing complication rates and longer hospitalization with higher ASA class among patients undergoing emergency surgery for acute cholecystitis. Nevertheless, other studies have shown acceptable outcomes in carefully selected patients with higher ASA scores (25). Taken together, these findings support considering ASA status as a marker of preoperative vulnerability, although its association with postoperative recovery may vary according to disease severity, case selection, and institutional perioperative capacity.

Tokyo Guidelines 2018 severity grade and time from symptom onset to surgery were included because both may confound associations between baseline patient characteristics and postoperative recovery. Neither remained associated with prolonged postoperative stay after multivariable adjustment. The estimate for symptom-onset-to-surgery time nevertheless remained compatible with a modest increase in risk per additional 24 h, although the confidence interval included the null value. These findings should not be interpreted as evidence that disease severity or timing is clinically unimportant; rather, their effects may overlap with age, ASA status, comorbidity burden, and other aspects of acute presentation. The admission-to-surgery interval was also characterized separately as a care-process measure. Because the primary outcome began at surgery and time from symptom onset to surgery was already included in the prespecified model, admission-to-surgery time was not entered simultaneously in the primary adjustment set to avoid conditioning on overlapping preoperative timing intervals.

Most procedures in this cohort began laparoscopically (92.2%), and 13.9% of laparoscopic starts required conversion to open surgery. Operative time and conversion were deliberately excluded from the primary explanatory model because they occur after preoperative assessment and may lie on the pathway between baseline disease characteristics and postoperative recovery. Previous studies have linked difficult laparoscopic cholecystectomy with longer operative times, conversion, and longer postoperative stay (23, 26). In the present study, these variables are therefore reported as perioperative characteristics rather than interpreted as independent preoperative determinants of length of stay.

Although population-based studies typically report a female predominance in acute cholecystitis (3), men represented 58.5% of the present surgical cohort. We rechecked the sex coding in the analytic dataset and found no evidence of category inversion or recoding error. This difference should be interpreted in light of the study population, which comprised patients undergoing cholecystectomy at a specialized emergency hospital rather than all individuals with acute cholecystitis in the community. Differences in referral patterns, disease presentation, operative selection, and institutional case mix may therefore contribute to the observed distribution, and the male predominance in this cohort should not be interpreted as contradicting the broader epidemiology of acute cholecystitis.

Postoperative infectious complications remained clinically relevant but were not considered explanatory variables in the revised primary model. Surgical site infection, pneumonia, and sepsis developed after surgery during the same admission in which postoperative length of stay was measured, making their temporal relationship with hospitalization inherently interdependent. Previous studies have similarly reported greater postoperative morbidity and longer postoperative stay in difficult laparoscopic cholecystectomy (26). Warren et al. (22) identified several patient and procedural characteristics associated with surgical site infection after cholecystectomy. Importantly, a recent systematic review and meta-analysis of randomized trials reported that antibiotic prophylaxis did not reduce postoperative infection in mild-to-moderate acute cholecystitis, underscoring that prevention of postoperative infection should not be reduced to antibiotic prophylaxis alone (27). We therefore report postoperative complications descriptively and avoid assigning a predictive or causal interpretation to their relationship with length of stay. Because the exact timing of complication onset or diagnosis was not consistently documented, we were also unable to determine whether individual complications occurred before or after postoperative day 3. This limitation further supports treating these events as descriptive postoperative outcomes rather than prognostic factors.

Hypertension and active smoking showed inverse adjusted associations with prolonged postoperative stay. These findings should not be interpreted as evidence of protective effects. Both associations may reflect residual confounding, differences in clinical selection or perioperative management, or limitations in retrospective measurement, particularly for smoking status. Because these findings were not hypothesized as protective relationships and require external replication, they should be regarded as exploratory observations rather than clinically actionable effects.

This study provides real-world evidence from a high-demand emergency hospital and has several strengths. The revised analysis included all 422 eligible patients rather than a sampled subset, used postoperative rather than total hospital stay as the primary outcome, incorporated Tokyo Guidelines 2018 severity and symptom duration, separated coexisting comorbidities, and prespecified the multivariable model according to clinical and temporal considerations. The low VIF values also provided no evidence of relevant multicollinearity. In addition, postoperative complications, mortality, and 30-day readmission were reported separately to avoid inappropriate temporal interpretation.

Nevertheless, several limitations should be considered. First, the retrospective design depends on the completeness and accuracy of routine medical records and does not establish causality. The exclusion of records with incomplete key timestamps, outcome information, or major covariate data may also have introduced selection bias if excluded patients differed systematically from those retained in the analytic cohort. Symptom-onset information was unavailable for 19 patients, resulting in a complete-case primary model of 403 patients. Second, the study was conducted at a single emergency hospital, and institutional admission, operative, and discharge practices may limit generalizability. Recent international evidence using cholecystectomy as a tracer procedure has shown marked between-hospital variation in surgical outcomes, including substantial differences that were not explained solely by national income level, emphasizing the importance of local systems and institutional capacity when extrapolating results (28).

Third, although inclusion of the full eligible cohort increased the number of patients in previously sparse strata, some subgroups remained relatively small, including patients classified as ASA IV–V (*n* = 38) and those who developed sepsis (*n* = 20). Sepsis and other postoperative complications were not included as explanatory variables in the primary model. In addition, the exact timing of postoperative complication onset or diagnosis was not consistently documented, preventing reliable classification of these events as occurring before or after the 3-day postoperative threshold. Postoperative complications were identified from diagnoses recorded during routine clinical care rather than independently adjudicated using standardized surveillance criteria, which may have introduced some degree of misclassification.

Finally, detailed laboratory trajectories, imaging-based measures of anatomical severity, and surgeon-level characteristics were not consistently available for the revised analysis. These unmeasured factors may have contributed to residual confounding. Accordingly, the adjusted associations should not be interpreted as fully independent clinical determinants of postoperative length of stay or as constituting a validated risk-prediction model.

In conclusion, prolonged postoperative hospital stay occurred in 37.4% of patients undergoing cholecystectomy for acute cholecystitis in this Peruvian emergency hospital. Older age, diabetes mellitus, and ASA IV–V status were associated with a higher risk of prolonged postoperative stay after adjustment for prespecified preoperative factors. The inverse associations observed for hypertension and active smoking should be interpreted cautiously and should not be considered protective effects. Postoperative complications were reported separately and were not interpreted as predictors of length of stay. These findings identify preoperative characteristics that warrant further evaluation in prospective, multicenter risk-prediction studies and should not be interpreted as evidence of causal effects or intervention effectiveness.

## Data Availability

The original contributions presented in the study are included in the article/Supplementary Material, further inquiries can be directed to the corresponding author.
