# Supplementary material for: Factors associated with prolonged postoperative hospital stay after cholecystectomy for acute cholecystitis in a Peruvian emergency hospital
Source: Front Surg. 2026 Oct 14;13:1921693. doi: 10.3389/fsurg.2026.1921693 (PMC13713639; doi:10.3389/fsurg.2026.1921693)
Supplement: Supplementary file 1 [file Supplementaryfile1.docx]

**Supplementary File S1. STROBE Checklist**

**STROBE Statement—Checklist of items that should be included in reports of cohort studies**

*Manuscript: Factors Associated With Prolonged Postoperative Hospital Stay After Cholecystectomy for Acute Cholecystitis in a Peruvian Emergency Hospital*

|  | Item No. | Recommendation | Reported on page/section of manuscript |
| --- | --- | --- | --- |
| Title and abstract | 1(a) | Indicate the study’s design with a commonly used term in the title or the abstract | Abstract. The Methods sentence identifies the study as a “retrospective cohort study.” |
| Title and abstract | 1(b) | Provide in the abstract an informative and balanced summary of what was done and what was found | Abstract. Objective, cohort design, source records, postoperative outcome definition, modified Poisson model, principal adjusted associations, and cautious conclusion are reported. |
| Background/rationale | 2 | Explain the scientific background and rationale for the investigation being reported | Introduction. Background covers acute cholecystitis burden, TG18 management, timing of surgery, postoperative recovery, and hospital resource use. |
| Objectives | 3 | State specific objectives, including any prespecified hypotheses | Introduction, final paragraph. Objective: determine preoperative factors associated with prolonged postoperative hospital stay after cholecystectomy. |
| Study design | 4 | Present key elements of study design early in the paper | Methods, Section 2.1. Retrospective cohort design, index hospitalization, and retrospective reconstruction from admission through surgery and discharge are stated. |
| Setting | 5 | Describe the setting, locations, and relevant dates, including periods of recruitment, exposure, follow-up, and data collection | Methods, Section 2.1. Hospital de Emergencias José Casimiro Ulloa, Lima, Peru; January–December 2024. Time zero for the primary analysis is surgery and the outcome period extends to discharge. |
| Participants | 6(a) | Give the eligibility criteria, and the sources and methods of selection of participants. Describe methods of follow-up | Methods, Sections 2.1–2.3. Source registry, screening of all 540 records, eligibility/exclusion criteria, and retrospective follow-up through discharge are described; Figure 1 shows participant flow. |
| Participants | 6(b) | For matched studies, give matching criteria and number of exposed and unexposed | Not applicable. This was not a matched cohort study. |
| Variables | 7 | Clearly define all outcomes, exposures, predictors, potential confounders, and effect modifiers. Give diagnostic criteria, if applicable | Methods, Section 2.4. Primary outcome, preoperative covariates, TG18 grade, symptom timing, comorbidities, intraoperative characteristics, postoperative complications, mortality, and readmission are defined. |
| Data sources/measurement | 8* | For each variable of interest, give sources of data and details of methods of assessment (measurement). Describe comparability of assessment methods if there is more than one group | Methods, Sections 2.4–2.5. Data were extracted from routine clinical records using a structured form; admission, symptom-onset, surgery, and discharge timestamps and clinical diagnoses are described. |
| Bias | 9 | Describe any efforts to address potential sources of bias | Methods, Sections 2.2, 2.4–2.6; Discussion, Limitations. All eligible records were analyzed, the adjustment set was prespecified, postoperative events were excluded from the primary explanatory model, no imputation was used, and selection/misclassification/residual confounding are acknowledged. |
| Study size | 10 | Explain how the study size was arrived at | Methods, Section 2.2. All 540 potentially eligible source records were screened; after 118 exclusions, all 422 eligible patients were included. No sampling procedure or sample-size calculation was used in the revised analysis. |
| Quantitative variables | 11 | Explain how quantitative variables were handled in the analyses. If applicable, describe which groupings were chosen and why | Methods, Sections 2.4 and 2.6. Age and ASA were categorized; obesity was BMI ≥30 kg/m²; TG18 grades were retained; symptom-to-surgery time was modeled per 24-h increase; prolonged postoperative stay was >3 days with a >4-day sensitivity definition. |
| Statistical methods | 12(a) | Describe all statistical methods, including those used to control for confounding | Methods, Section 2.6. Modified Poisson regression with robust variance, crude and adjusted RRs, prespecified clinically/temporally grounded adjustment set, VIF assessment, and software are described. |
| Statistical methods | 12(b) | Describe any methods used to examine subgroups and interactions | Not applicable. No subgroup or interaction analyses were performed. |
| Statistical methods | 12(c) | Explain how missing data were addressed | Methods, Section 2.6. Symptom-onset data were unavailable for 19 patients; the primary model used complete cases (n=403), with no imputation. |
| Statistical methods | 12(d) | If applicable, explain how loss to follow-up was addressed | Index-hospitalization follow-up was reconstructed through discharge and therefore no loss to follow-up occurred for the primary outcome among the 422 eligible patients. Thirty-day readmission was evaluated among 417 patients discharged alive. |
| Statistical methods | 12(e) | Describe any sensitivity analyses | Methods, Section 2.6; Results; Supplementary Table S1. The prespecified model was repeated using postoperative stay >4 days to assess robustness to the >3-day threshold. |
| Participants | 13(a) | Report numbers of individuals at each stage of study—e.g., numbers potentially eligible, examined for eligibility, confirmed eligible, included in the study, completing follow-up, and analysed | Results; Figure 1. 540 source records screened, 118 excluded, 422 eligible/included, and 403 complete cases in the primary multivariable model. |
| Participants | 13(b) | Give reasons for non-participation at each stage | Methods, Section 2.2; Figure 1. Reasons and counts are reported for all 118 exclusions. |
| Participants | 13(c) | Consider use of a flow diagram | Figure 1. STROBE participant flow diagram included. |
| Descriptive data | 14(a) | Give characteristics of study participants (e.g., demographic, clinical, social) and information on exposures and potential confounders | Results; Table 1. Demographic, comorbidity, ASA, TG18 severity, symptom timing, operative approach, conversion, and operative-time characteristics are reported. |
| Descriptive data | 14(b) | Indicate number of participants with missing data for each variable of interest | Methods, Section 2.6; Results; Table 1; Figure 1. Symptom-onset data were missing for 19/422 (4.5%); the primary outcome and main retained variables were available for the eligible cohort. |
| Descriptive data | 14(c) | Summarise follow-up time (e.g., average and total amount) | Results; Table 1. Postoperative follow-up from surgery to discharge is summarized by postoperative stay: median 2.4 days (IQR 1.0–3.8). Total stay and admission-to-surgery interval are also reported. |
| Outcome data | 15* | Report numbers of outcome events or summary measures over time | Results; Tables 1–2. Prolonged postoperative stay occurred in 158/422 (37.4%); postoperative complications, mortality, and 30-day readmission are reported descriptively. |
| Main results | 16(a) | Give unadjusted estimates and, if applicable, confounder-adjusted estimates and their precision (e.g., 95% confidence interval). Make clear which confounders were adjusted for and why they were included | Results; Table 3; Figure 2. Crude and adjusted RRs with 95% CIs are reported; Methods explains the clinically and temporally prespecified adjustment set. |
| Main results | 16(b) | Report category boundaries when continuous variables were categorized | Methods, Section 2.4; Table 1. Age groups, obesity threshold (BMI ≥30 kg/m²), ASA categories, TG18 grades, and postoperative-stay thresholds are explicitly stated. |
| Main results | 16(c) | If relevant, consider translating estimates of relative risk into absolute risk for a meaningful time period | Results and Table 1 report the absolute outcome frequency directly: 158/422 patients (37.4%) had postoperative stay >3 days during the index hospitalization. |
| Other analyses | 17 | Report other analyses done—e.g., analyses of subgroups and interactions, and sensitivity analyses | Results; Supplementary Table S1. Sensitivity analysis using postoperative stay >4 days is reported; no subgroup or interaction analyses were performed. |
| Key results | 18 | Summarise key results with reference to study objectives | Discussion, first paragraph. Principal adjusted associations and the temporal handling of intraoperative/postoperative factors are summarized in relation to the study objective. |
| Limitations | 19 | Discuss limitations of the study, taking into account sources of potential bias or imprecision. Discuss both direction and magnitude of any potential bias | Discussion, Limitations. Retrospective measurement, selection bias from incomplete records, missing symptom-onset data, complication timing/misclassification, sparse strata, single-center generalizability, unmeasured laboratory/imaging/surgeon factors, and residual confounding are discussed. |
| Interpretation | 20 | Give a cautious overall interpretation of results considering objectives, limitations, multiplicity of analyses, results from similar studies, and other relevant evidence | Discussion and Conclusion. Findings are interpreted as associations rather than causal effects; inverse associations are explicitly treated cautiously; sensitivity results and prior literature are considered. |
| Generalisability | 21 | Discuss the generalisability (external validity) of the study results | Discussion, Limitations. Single-emergency-hospital setting and institutional variation are discussed, with international evidence used to contextualize external validity. |
| Funding | 22 | Give the source of funding and the role of the funders for the present study and, if applicable, for the original study on which the present article is based | Funding section. The authors state that no financial support was received; therefore, there was no funder role in the study. |
